# The impact of digital industry development on regional economic resilience: Evidence from China

**DOI:** 10.1371/journal.pone.0315203

**Published:** 2025-02-19

**Authors:** Sui Tian, Yiran He

**Affiliations:** College of Economics, Hangzhou Dianzi University, Hangzhou, Zhejiang, China; Alexandru Ioan Cuza University of Iasi, Faculty of Philosophy and Social-Political Sciences, ROMANIA

## Abstract

With the outbreak of the global public health crisis in 2019, enhancing the resilience of regional economies has become the current focal point. Existing studies have mostly focused on the region itself, lacking exploration of regional economic resilience from the aspects of dynamics, multiple perspectives, and multidimensional integration. At the same time, the digital industry, as an emerging sector, should not only consider its impact on economic development itself, but also focus on whether it can continuously and effectively enhance the level of regional economic resilience, in order to cope with crises that may arise at any time. Therefore, through empirical methods, we conducted a detailed study of the spatial correlation and internal driving factors between the digital industry and regional economic resilience, aiming to build a more valuable theoretical framework based on existing research findings and explore a regional resilience development strategy centered around the digital industry. This paper, combining conclusions and methods from existing literature, attempts to expand the definition of regional economic resilience, evaluation index system, and the relationship with the digital industry from the perspective of evolutionary economic geography. This article empirically examines data from 30 provinces in China from 2014 to 2022 (excluding Tibet, Hong Kong, Macau, and Taiwan due to lack of data). Firstly, this paper employs a two-way fixed effects model to examine the direct relationship between digital industry development and regional economic resilience. The research results indicate that the development of the digital industry can effectively enhance regional economic resilience. Secondly, the role of spatial location, as an important aspect of evolutionary economic geography, is also considered in this paper. The spatial Durbin model is used to discover spatial spillover effects of digital industry development on regional economic resilience under different spatial location relationships. Finally, this paper considers environmental regulations as a threshold variable to study the impact of the digital industry on regional economic resilience under different levels of environmental regulation. The results indicate that when the degree of environmental regulation is less than 0.0011, the digital industry can more effectively empower the enhancement of regional economic resilience levels. In conclusion, this paper finds that while emphasizing the role of the digital industry in the resilient development of regional economies, it is also essential to promote regional cooperation for mutual benefit and win-win results. This will accelerate the transformation of digital enterprises, optimize industrial structures, and achieve green development.

## 1. Introduction

Similar to other fields in the social sciences, resilience has become a crucial topic for economic geographers studying how economic systems withstand and recover from unforeseen events. Over the past few decades, with the accelerating pace of global integration and the frequent occurrence of global crises, the external shocks and disaster risks faced by various countries and regions have been gradually increasing, and this trend is expected to continue. This situation arises primarily due to unforeseen events such as geopolitical conflicts, natural disasters, and public health crises, triggering widespread and profound social unrest. So far, many scholars in economic geography have drawn insights from engineering and ecology regarding system equilibrium and have metaphorically constructed the concept of ’regional economic resilience’ [1]. The purpose of this metaphor is to endow the economic entity with sufficient resistance and resilience when facing unpredictable external shocks, allowing it to quickly recover from crises and return to a normal developmental trajectory. Especially with the sudden outbreak of the public health crisis in 2019, governments worldwide began to highly prioritize the construction of regional economic resilience to better cope with the constantly changing and complex external environment. However, the development of the digital industry, as a major driver of economic growth, has become a key pathway to enhance regional economic resilience. In the tide of technological development, there is a continuous emergence of innovative technologies, gradually perfecting the entire industry chain. Digital transformation has brought about new business strategies, promoted job creation, and created growth opportunities for regional economies. Meanwhile, the widespread application of digital technology has not only increased production efficiency but also driven optimal resource allocation, further strengthening the resilience of regional economies. With the advent of the digital era, regional economies have become more vibrant, demonstrating robust vitality in the face of various future challenges.

The digital economy refers to a series of economic activities where data resources serve as key production factors, modern information networks act as important carriers, and the effective use of information and communication technology serves as a crucial driver for efficiency enhancement and economic structure optimization. With the prosperity of the global digital economy, scholars’ perspectives on how the digital economy enhances the resilience of regional economies have gradually gained widespread attention. The relevant analysis not only focuses on short-term coping strategies for regions facing external shocks but also delves deeper into how the development of the digital economy can adjust economic systems to achieve sustainable development [2, 3]. At the same time, the digital industries that have gradually differentiated from the digital economy, as new industries for economic strategic development and the core for unleashing economic vitality, also play a manifest role in enhancing regional economic resilience. The digital industry refers to economic activities that provide digital technologies, products, services, infrastructure, and solutions for the digital development of industries, as well as those entirely reliant on digital technologies and data elements. Of note, the development of the digital economy is a constantly changing process that encompasses both ’innovative’ and ’disruptive’ aspects [4]. Therefore, some scholars propose that the development of the digital economy may have a negative impact on economic resilience, urging to avoid a situation where blind development of the digital economy leads to a decrease in economic resilience [5]. In this process, there are also some pressing challenges that need to be addressed and overcome. Firstly, there is still a significant amount of controversy surrounding the concept of economic resilience at the current stage [6]. Over time, this concept has undergone multiple revisions [7]. Secondly, although many documents theoretically elaborate on how the digital economy can enhance economic resilience [8], there are few scholars who conduct in-depth research on the relationship between the digital industry and economic resilience.

The following article aims to provide a beneficial expansion of the concept of economic resilience from the perspective of evolutionary economic geography, and based on this, to construct an index system that conforms to the regional economic resilience in China. Building upon previous research findings, this paper utilizes panel data from 30 provinces in China from 2014 to 2022. Initially, it conducts a two-way fixed-effects regression to examine the direct impact of digital industry development on regional economic resilience, bolstering the reliability of the experimental results through robustness and endogeneity tests. Secondly, within the analytical framework of evolutionary economic geography, locational space plays an indispensable role in the study of regional economic resilience. Hence, this paper employs a spatial panel model to examine the relationship between the direct, indirect, and total effects of digital industry development on other aspects of regional economic resilience. Lastly, the integration of policy and evolution has always been a topic of discussion in evolutionary economic geography. China has consistently emphasized placing ecological conservation in a more prominent position, thus implementing strict environmental regulatory measures to improve the ecological environment. Therefore, this article discusses the regulatory role of environmental regulations in the growth of the digital industry and the study of regional economic resilience, namely the indirect impacts, and conducts an empirical study using threshold regression. Based on the specific conditions of regional development, it provides valuable policy recommendations to enhance the resilience of the regional economy.

## 2. Literature review and mechanism analysis

### 2.1. The concept and measurement of resilience

Holling proposed the concept of ‘resilience’ and defined it as ‘engineering resilience’ [9]. Today, this concept has been widely applied in the social and economic fields [10]. According to its strict Latin root, ’resilire,’ it refers to the ability to recover and restore after being impacted, specifically referring to the action of rebounding or jumping [11]. When we incorporate a concept into the theoretical framework of economics, it manifests as a very traditional and widely used ’engineering resilience.’ This resilience implies that when an economy is impacted and forced to deviate from its original position and trajectory, the system is capable of returning to its original equilibrium state and maintaining its stability [12]. ’Ecological resilience’ is another description of resilience, referring to the system’s ability to absorb impacts while maintaining its structure, functionality, and characteristics unchanged [13], which Holling defined as ’ecological resilience. Economic geographers holding the perspective of ecological resilience point out that although economic entities experience only a brief moment of shock in their growth trajectory, it has far-reaching implications for their future development patterns and evolutionary trajectories. Regardless of whether viewed from the perspective of ’engineering resilience’ or ’ecological resilience,’ the focus is on the level of recovery ability and resilience after being impacted. The primary measurement methods usually describe this by observing the continuous dynamic changes of a specific indicator over time before and after the shock, which has certain limitations. However, guided by equilibrium thinking, both ’engineering resilience’ and ’ecological resilience’ have also faced some degree of criticism in their development trajectories [14, 15]. Evolutionary economic geographers argue that resilience is an inherent attribute of a region. They believe that the resilience of regional economies is not just a specific static attribute within that region but also a direct product of its continuous development and change. Especially in the case of facing shocks, this resilience may become even more significant. From an evolutionary perspective, the resilience of regional economies is redefined as the ability to continuously create new paths for growth within the region [16]. This implies that regional economies can achieve self-recovery, renewal, and transformation through the reorganization of various elements [17]. Therefore, within this theoretical framework, economic resilience is typically measured by constructing an evaluation indicator system [18–20].

### 2.2.The concept and measurement of the digital industry

The concept of digital industries is generally considered a term used primarily within Chinese academia and industry, reflecting a certain regional specificity [21]. In China, the concept of the digital economy can evolve into two related concepts: digital industrialization and industrial digitization. Comparing similar concepts can help better understand digital industrialization and the significance of its introduction in China. In Germany, the term ‘Industrie 4.0’ is widely used, while in the United States, terms like ‘IoT’ or ‘digital manufacturing’ are more prevalent [22]. Initially, the concept of ‘Industrie 4.0’ can be traced back to 2011, when German professor Wolfgang Wahlster introduced it at the Hannover Messe. It involves the digital transformation of manufacturing through the convergence of technologies such as big data, cloud computing, digital twins, IoT, and artificial intelligence/machine learning [23]. In other words, it combines advanced digital technologies with industrial machinery processes to enhance operational efficiency, productivity, and automation as much as possible. ‘Industry 4.0’ is most notably represented by the use of smart factories as a key element in improving the efficiency of parts production, on which a series of new production methods are built. Of course, this concept is based on the enormous innovative driving force of digital technology [24]. However, in China, the concept analogous to ‘Industry 4.0’ is more akin to industrial digitization rather than digital industrialization [25]. Industrial digitization means the comprehensive transformation of traditional industries through digital technology to promote the deep integration of digital technology with the real economy [26], thereby enhancing the total factor productivity of the real economy [27]. This not only focuses on the digital transformation of traditional manufacturing [28, 29] but also extends to the digitization of cultural industries [30, 31], service industries [32, 33], and more. Digital industrialization, on the other hand, refers to providing digital technologies, products, services, infrastructure, and solutions for the development of industrial digitization, as well as various economic activities that are entirely dependent on digital technologies and data elements.

After clarifying the relevant concepts, let’s discuss why China has introduced the concept of digital industrialization, which has regional characteristics. With the continuous advancement and widespread application of digital technology, various social production activities can now be transformed into recordable, storable, and interactive data, information, and knowledge through digital means, turning data into a new production resource and key production factor. As advanced network technologies like the Internet and the Internet of Things continue to progress and be widely applied, we can observe the flow, connection, and integration of abstract data, information, and knowledge between different entities. This process profoundly reshapes traditional production modes and production relationships. With the continuous advancement of artificial intelligence technologies and the widespread application of advanced data information processing and communication technologies such as information systems, big data, cloud computing, and quantum communication, the efficiency and capability of data processing have significantly improved. The application of these technologies has greatly enhanced the timeliness, automation, and intelligence of data processing, thereby driving the efficiency of social and economic activities and the rapid growth of social productivity. Against this backdrop, the development of China’s digital industry has become a new growth point for economic development and has had a significant impact on national economic growth. Therefore, China places great importance on the development of the digital industry. The National Bureau of Statistics of China’s Order No. 33 document, ’Statistical Classification of Digital Economy and Its Core Industries (2021),’ [34] provides a detailed explanation of the statistical classification of the digital industry. This includes core sub-industries such as software and information technology services, internet and service industries, telecommunications, and electronic information manufacturing, representing the development direction and application results of the new generation of digital technologies.

### 2.3. The role of digital industrialization in resilience

Economic growth can be considered a process of ’innovative disruption,’ in which new economic activities continually emerge while traditional economic activities gradually disappear [35]. From a particular perspective, evolutionary economic geography views regional development as a continuous process, with emerging industries constantly arising, and traditional industries gradually declining. The digital industry primarily encompasses core subsectors such as software and information technology services, internet and related services, telecommunications, and electronic information manufacturing. As an emerging field, it not only reflects the current characteristics of digital economic development but also represents the future direction and application prospects of the next generation of digital technologies [36]. As a burgeoning sector, the digital industry’s role in shaping the additional paths of regional development has become a central issue in understanding regional economic resilience [37]. On one hand, the digital industry inherently exhibits characteristics of technological concentration, deep penetration, and pioneering nature [38]. Through the innovation and application of digital technology, we can derive new industries and chains from existing industries and fields, not only enhancing the diversity and heterogeneity of related industries but also opening up new pathways to improve the resilience of regional economies. From another perspective, advancing the development of the digital economy has become a crucial strategic choice for capturing new opportunities in the wave of technological change and industrial transformation. The global layout and actual implementation of digital economic strategies proceed simultaneously, and the development strategy of digitalization consistently influences the decision-making directions of entrepreneurs and governments. The digital industry is regarded as the core support for the development of the digital economy. Therefore, targeted digital industry strategic actions by entrepreneurs and governments can pave the way for new paths, thereby enhancing the resilience of the regional economy. Therefore, the following hypothesis is proposed in this paper:

H1: The development of the digital industry can effectively empower the improvement of regional economic resilience

### 2.4. The function of spatial location

Within the theoretical framework of evolutionary economic geography, spatial location plays an undeniable role in the study of digital industrialization and regional economic resilience [39]. Firstly, under the influence of geographical proximity, there is a phenomenon of knowledge spillover among enterprises, especially in the transfer of tacit knowledge. The diffusion of tacit knowledge not only requires face-to-face learning but also relies more on geographical proximity [35]. Therefore, leveraging the spillover effect of knowledge, digital enterprises have adopted innovative management strategies and technological means, opening up new paths for regional development and enhancing the economic resilience of the area. Furthermore, under the influence of economic distance, enterprises are more likely to form cognitive adjacency relationships, leading to the emergence of network effects [40]. In situations where the level of regional economic development is similar, digital enterprises have a closer understanding of development, and communication between enterprises becomes more frequent and fluent, thereby contributing to the improvement of the region’s economic resilience level. Lastly, considering geographical distance, digital enterprises differ from traditional enterprises, as their ability to circulate data is enhanced, contributing to breaking information isolation and industry barriers, thus promoting the integration and connection of high-value data [41]. Although digital enterprises may have certain geographical distances, they can achieve the social configuration of data elements among various industries and departments, thus enhancing the resilience of regional economies through the mutual connection and exchange of data elements. At the same time, we must also pay attention to the ’siphon effect’ exhibited by spatial spillover, which is detrimental to the economic resilience of surrounding areas [42]. The main reason is that the cross-regional flow of resources tends to shift towards developed regions with higher production efficiency and capital returns, creating a ’siphon effect’ in the resource-absorbing developed regions, which in turn reduces the economic resilience of the surrounding areas. Additionally, low-cost learning effects and imitation effects may lead to resource wastage, causing a certain degree of negative externalities on the surrounding regions [43]. Therefore, we propose the following hypothesis:

H2a: The development of the digital industry has a positive spatial spillover effect on regional economic resilience.
H2b: The development of the digital industry exerts a negative spatial spillover effect on regional economic resilience.

### 2.5. A new perspective on environmental regulation

Although researchers in evolutionary economic geography have recognized the central role of policy and institutions in their studies, there is relatively little in-depth research on how specific policies affect dynamic evolution [44]. Environmental regulation, as a socially driven regulation promoted by the government, aims to encourage businesses to reduce environmental pollution in their production and operational activities, and promote the harmonious development of the ecological environment and economy, which is a key factor influencing the resilience of the digital industry to regional economies. With the continuous strengthening of local environmental regulations, businesses face stricter government oversight, which also means they need to pay higher pollution emission costs [45]. As a result of rising business costs, some companies also face increased production expenses, further weakening their competitive advantage in the market. Some small-scale businesses may face survival pressure, need to lay off employees or reduce investments, which undoubtedly has a negative impact on local employment opportunities and economic vitality, casting a shadow over regional economies. Although the digital industry may not appear to be a high-energy-consuming or highly polluting sector on the surface, in reality, many manufacturers have relatively backward technology and equipment, and their industrial structure is not very rational, leading to high energy consumption and environmental pollution issues. Therefore, the cost of pollution control in the digital industry increases, thereby reducing the impact of the digital industry on regional economic resilience. Conversely, strict environmental regulation policies can lead to a decrease in the negative externality of environmental pollution, thereby transforming the positive externality of environmental improvement into economic benefits. The main reason is that the reduction of environmental pollution may increase the accumulation of human capital [46], leading to an increase in urban scale and population concentration, thereby enhancing the innovation capability of the digital industry and increasing its impact on regional economic resilience. Therefore, the following hypothesis is proposed in this paper:

H3: The impact of digital industry development on regional economic resilience is subject to a threshold effect of environmental regulation. When environmental regulation is at a lower level, the effect of digital industry development on enhancing regional economic resilience is stronger; when environmental regulation is at a higher level, the effect is weaker.

## 3. Methodology

This method mainly includes four stages: The first stage involves constructing indicators for regional economic resilience. Using data from 30 provinces and cities in China from 2014 to 2022, this paper develops an indicator system (data sourced from the National Bureau of Statistics of China, CNRDS, and CEIC databases). The Stata software is used, and the entropy weight method is employed to derive specific values, which is a commonly used multi-attribute decision analysis method for evaluating the advantages and disadvantages of alternative options. It combines the entropy method and the TOPSIS method to objectively determine indicator weights, reducing the influence of subjective factors. The second phase involves testing the impact of the digital industry on regional economic resilience. This study constructs an economic model and uses Stata software to empirically verify the bidirectional fixed effects, aiming to explore how the development of the digital industry affects regional economic resilience and whether H1 aligns with expectations. The third step examines whether the digital industry has spatial spillover effects on regional economic resilience when considering different spatial weights. This paper tests this through Moran’s I and spatial detection models, constructing a Spatial Durbin Model and using Stata software to verify whether H2 aligns with expectations. The fourth step involves investigating whether there is a threshold effect of environmental regulation on the impact of the digital industry on regional economic resilience. A double threshold model is constructed, and Stata software is used to verify whether H3 aligns with expectations. The specific steps are as follows.The moral statement does not apply here.

### 3.1. Variable description and construction of the indicator system

#### 3.1.1. Framework and analysis of the dependent variable indicator system

This article conceptualizes the concept of regional economic resilience based on the idea of evolutionary economic geography, as the ability of the economic system to respond to short-term shocks and long-term development in the future. China’s regional economic resilience is defined based on evolutionary economic geography and is formed on the basis of China’s establishment of a new development pattern of dual circulation. China’s regional economic resilience is mainly composed of six factors: material foundation, technological innovation, market overview, structural optimization, social security, and opening up, forming a complex entity [47]. Referring to existing research [48–50], the material foundation, market overview, and social security among these six factors are classified as short-term coping abilities of resilience and recovery, and adaptation and adjustment. At the same time, technological innovation, structural optimization, and opening up are classified as long-term development capabilities of innovation transformation and structural resilience. The former focuses on short-term social coping abilities under impact, while the latter focuses on long-term social development capabilities. Our focus here is on developing a method to assess regional economic resilience, referencing the concept of regional economic resilience in this article. We use panel data from 30 provinces in China from 2014 to 2022 (data from Tibet, Hong Kong, Macao, and Taiwan are severely lacking). We quantify the index system using the entropy weighting method to characterize the recovery, adjustment, transformation, and resilience of the economic system to disturbances. The data is sourced from the China Statistical Yearbook over the years, with missing data supplemented by CNRDS and CEIC databases. The specific index construction system is shown in Table 1:

**Table 1:** Regional economic resilience indicator system.

Primary Indicator	Secondary Indicators	Three-level indicator
Resistance resilience	Per Capita GDP	Regional GDP growth rate
	Urban registered unemployment rate	Urban Unemployment Rate/Total Population
	Per Capita Disposable Income	Per capita disposable income in the region
	Internet penetration rate	Number of Internet broadband access users/permanent residents
Adaptability and adjustment strength	Public Welfare-Oriented Fiscal Expenditure	The proportion of local fiscal budget expenditures on housing, healthcare, education, social security, and employment
	Proportion of deposits and loans in financial institutions	Deposit Amount of Financial Institutions / Loan Amount of Financial Institutions
	Fiscal self-sufficiency ratio	Revenue / Expenditure
	Total social fixed asset investment	Regional Fixed Asset Investment
Innovative Transformation Power	Research and development investment intensity	Research and Development (R&D) expenditure of industrial enterprises above a certain scale as a percentage of GDP
	Technical Trading Activity	Technical transaction turnover/GDP
	Investment Efficiency	Investment Rate/Regional GDP Growth Rate
	Number of granted patents	Number of authorized patents in the region
Structural Bearing Capacity	Demand Structure	Retail Sales of Consumer Goods/GDP
	Urban-Rural Structure	Urbanization Rate
	Foreign Trade Dependency	Total Import and Export/Gross Domestic Product (GDP)
	Government Debt Burden	Government debt balance/GDP

Firstly, when considering resilience against risks, the focus is on the overall economic environment at a short-term point when faced with sudden risks. Per capita GDP, as an important indicator of a country or region’s economic development, has a strong indicative significance for short-term economic operations [51]. The urban registered unemployment rate is regarded as one of the key indicators for evaluating employment conditions and social stability. When a region encounters sudden risks, the higher the number of unemployed, the less stable society becomes, thus increasing the risk [52]. Per capita disposable income is an important measure of residents’ living standards, and in the face of short-term risks, regions with higher living standards tend to have stronger resilience and faster recovery times [53]. The internet penetration rate is a key indicator of a nation’s level of informatization, directly affecting economic and social development. With the widespread use of the internet, people’s awareness of risks increases when faced with challenges, providing more ways to understand these risks [54]. The above are all macro-level indicators that affect the overall economic environment.

Secondly, the regulation of adaptability mainly considers the ability of short-term government aid and social capital replenishment in times of crisis. Livelihood-related fiscal expenditure, as an important part of public finance, has played a positive role in ensuring and improving people’s basic living conditions and promoting social harmony and stability. In times of crisis, the larger the livelihood-related fiscal expenditure, the higher the level of protection, which can provide sufficient momentum and support for economic development [55]. The proportion of loans and deposits in financial institutions, as an important part of the balance sheet, is a key indicator of the bank’s operational status and risk level. In times of crisis, commercial banks need to adjust their credit structures in a timely manner, increase support for the real economy sectors such as enterprises and residents, while also preventing capital idling. The self-sufficiency rate of government finances, as an important indicator reflecting the scale and structure of government revenue and expenditure, holds a crucial position in China’s public finance system. In times of crisis, the self-sufficiency rate can provide timely warnings and adjust the direction of fiscal expenditure policies [56]. The total investment in fixed assets by the whole society, as an important macroeconomic indicator, has significant importance for analyzing the state of the national economy and predicting economic trends [57]. In times of crisis, enterprises are seeking more effective investment methods to improve economic efficiency. The above are all macro-level indicators for managing capital flows in response to crises.

Re-examining the power of innovation and transformation primarily considers the level at which regions accelerate long-term development after facing a crisis. R&D intensity, as a key component of innovation input, has a significantly positive motivating effect on corporate technological innovation [58]. After facing a crisis, it can further strengthen corporate competitiveness, enhance competitiveness, and stimulate innovative vitality. The activity level of technology transactions, as a crucial indicator of the efficiency of corporate technological innovation, holds great significance for promoting industrial structure adjustment and upgrading [59]. After facing a crisis, the activity level of technology transactions plays a certain role in promoting the construction of the national innovation system and accelerating regional economic transformation and upgrading. Investment efficiency, as an important indicator in modern economic activities, is a core measure of corporate economic performance. After facing a crisis, improving investment efficiency is crucial for regional economic resilience and sustainable development. The number of patent authorizations, as an important indicator of corporate innovation capability, to some extent reflects the strength of corporate innovation. After facing a crisis, the number of patent authorizations has a strong guiding effect on regional technological development. All of the above are indicators of how innovation capacity enhancement leads regional recovery after a crisis.

Finally, the structural resilience mainly considers the long-term impact of the structural system on regional economic resilience. This paper measures structural resilience using demand structure, urban-rural structure, dependence on foreign trade, and government debt burden. Demand structure reflects the structural characteristics of China’s economy; urban-rural structure represents the spatial pattern of urban development and is a comprehensive reflection of the relationship between economic and social development levels and the rural situation [60]; dependence on foreign trade reflects a country’s international status and is an important indicator of a nation’s comprehensive strength and economic power; and government debt burden reflects the financial condition of local governments at all levels, serving as an important indicator of regional fiscal strength and public service levels. The above analysis examines China’s regional economic structure and form in responding to crises from four dimensions.

The method for calculating Regional Economic Resilience (RER) based on the entropy weight assignment method is as follows.

Based on data from 270 indicators in 30 provinces of China from 2014 to 2022 (excluding Tibet, Hong Kong, Macau, and Taiwan), an evaluation matrix X was established. Here, the *x*_*mn*_ in the matrix represents the numerical value of the n indicator of the m data point.


$$X=[\begin{array}{cccc} x_{11} & x_{12} & \ldots & x_{1n}\\ x_{21} & x_{22} & \ldots & x_{2n}\\ \vdots & \vdots & \ldots & \vdots \\ x_{m1} & x_{m2} & \ldots & x_{mn} \end{array}](m=30,n=16)$$

The evaluation matrix X is standardized to obtain the standardized matrix Y, where each element *y*_*ij*_ in Y corresponds one-to-one with *x*_*mn*_ in the evaluation matrix X. The standardization formula used in this paper is:

$$y_{ij}=[\frac{x_{mn}-\min\;x_{mn}}{\max\;x_{mn}-\min\;x_{mn}}]\times 100$$

The calculation yields the entropy value *E* = {*e*_1_,*e*_2_,⋯,*e*_*n*_} for each indicator in the standardized matrix Y and the corresponding entropy weight *W* = {*w*_1_,*w*_2_,⋯,*w*_*n*_}. The formulas for calculating entropy value and entropy weight are as follows:

$$e_{j}=\{\begin{array}{l} -k\sum_{i=1}^{n}\frac{y_{ij}}{\sum_{i=1}^{n}y_{ij}}\ln(\frac{y_{ij}}{\sum_{i=1}^{n}y_{ij}})(y_{ij}\neq 0)\\ \;0\;(y_{ij}=0) \end{array}(k=\frac{1}{\ln(m)}>0,m=270)$$

$$w_{j}=\frac{1-e_{j}}{n-\sum_{j=1}^{m}e_{j}}(n=16)$$

After multiplying the entropy weight with the corresponding standardized indicators and summing them horizontally, the digitalization level index *D* = {*d*_1_,*d*_2_,⋯,*d*_*n*_} can be calculated. The formula for calculating the digitalization level index of the i data is:

$$d_{j}=\sum_{j=1}^{n}w_{i}y_{ij}$$

#### 3.1.2. Explanation and analysis of independent variables

This article argues that measuring digital industrialization should start from the perspective of enterprises. Only by clarifying micro-mechanisms can one macroscopically control the logic of promoting high-quality and sustainable economic development through digital industries [61]. The measurement method for the digital industry (DIG) is: firstly, selecting the annually added Digital industry companies from the national industrial and commercial enterprise registration data. This mainly includes computer communication and other electronic equipment manufacturing, telecommunications, radio, television, and satellite transmission services, the internet and related services, software, and information technology services. Then, based on the location of Digital industry companies, the annual increase in the number of digital economic enterprises in each province is determined, representing the level of digital industrialization. As the remaining data are derived from ratios, this article uses logarithmic processing for the level of digitalization to ensure the accuracy of regression results.

#### 3.1.3. Explanation and analysis of threshold variables

Environmental regulations are rules established by the government for environmental protection, and they are a universal requirement that can impose green restrictions on business practices [62]. In the past, due to the need for economic development, environmental regulation policies in China were relatively lenient. However, in recent decades, with social progress and changes in economic growth patterns, environmental protection has gradually become a crucial issue in China’s development path. As the level of environmental regulation increases, and the government imposes higher environmental protection requirements on enterprises, it inevitably leads to changes in the role of the digital industry in regional economic resilience [63]. Therefore, this paper uses environmental regulation (ENV) as a threshold variable to study the impact of digital industry development on regional economic resilience under different levels of environmental regulation. The measurement involves the ratio of investment completed in industrial pollution control to industrial added value in each province, with data sourced from the statistical yearbooks of respective provinces.

#### 3.1.4. Explanation and analysis of control variables

The control variables selected in this article include: Traffic Accessibility (TRA), expressed as the ratio of regional road kilometers to regional area, with data from the Statistical Yearbook of each province; Energy Consumption (ENE), represented by GDP energy consumption per unit, sourced from the China Energy Statistical Yearbook; Industrial Structure (IST), indicated by the ratio of the tertiary industry to the secondary industry, with data from the China Statistical Yearbook; Marketization Level (MAR), sourced from the Marketization Index Report. Traffic accessibility, as a control variable, reflects the dynamic changes in traffic conditions and is an important indicator of urban infrastructure development levels. It impacts regional economic resilience in the short term by influencing risk rescue capacity. Energy consumption, as a critical indicator of regional economic development, partially reflects whether a region meets reasonable requirements for resource utilization and environmental protection. It affects regional economic resilience in the short term by influencing recovery resistance capacity. Industrial structure, the foundation of a country or region’s economic development, is determined by productivity and production relations. It impacts regional economic resilience in the long term by influencing structural endurance capacity. The level of marketization, an important measure of enterprise competitiveness, plays a crucial role in improving the quality of economic development in China. It impacts regional economic resilience in the long term by influencing innovation transformation capacity [64].

### 3.2. Benchmark model

Based on the existing research methods in the academic field [65], this paper constructs a regression model covering the digital industrialization and regional economic resilience:

$$RER_{it}=a_{0}+a_{1}DIG_{it}+a_{x}Z_{it}+\mu_{i}+d_{t}+\varepsilon_{it}$$

where RER is the explained variable, i.e., regional economic resilience. DIG is the explanatory variable: digital industrialization. Z is the set of control variables; μ represents individual effects, d represents time effects and ε is the random disturbance term.

### 3.3. Spatial econometric model

#### 3.3.1. Space weight configuration

To verify the spatial effects of the digital industry’s development on regional economic resilience, this paper constructs adjacency weight matrix, distance weight matrix, and economic weight matrix. The specific matrix forms are as follows:

1. Adjacency Weight Matrix. Based on the spatial adjacency of regions, when regions *i* and *j* are not adjacent, the value of *W*_1_ is 1; when regions *i* and *j* are adjacent, the value of *W*_1_ is 0.
$$W_{1}=\{\begin{array}{l} 0\;If\;i\;and\;j\;are\;not\;adjacent\\ 1\;If\;i\;is\;adjacent\;to\;j \end{array}$$
2. Distance Weight Matrix. Consider the impact of inter-regional distance. When region *i* and region *j* are the same, the distance matrix is 0; when region *i* and region *j* are different, the reciprocal of the distance between regional capitals is used as the distance weight matrix. *d*_*ij*_ representing the distance between provincial capitals measured by latitude and longitude.
$$W_{2}=\{\begin{array}{l} 0\;(i=j)\\ 1/d_{ij}\;(i\neq j) \end{array}$$
3. Economic Weight Matrix. Based on the reciprocal of the difference in per capita GDP between regions, taking into account the economic development situation.
$$W_{3}=\{\begin{array}{l} 0\;(i=j)\\ 1/|perGDP_{i}-perGDP_{j}|(i\neq j) \end{array}$$

#### 3.3.2. Spatial autocorrelation test

To examine whether regional carbon emissions exhibit spatial correlation, this paper employs the global Moran’s index for validation, with the corresponding calculation formula as follows: $Moran's\;I=\frac{\sum_{i=1}^{n}\sum_{j=1}^{n}W(Y_{i}-\bar{Y})(Y_{j}-\bar{Y})}{S^{2}\sum_{i=1}^{n}\sum_{j=1}^{n}W}$

Among them: $S^{2}=\frac{1}{n}\sum_{i=1}^{n}(Y_{i}-\bar{Y})$; $\bar{Y}=\frac{1}{n}\sum_{i=1}^{n}Y_{i}$ *Y*_*i*_ is the regional economic resilience of the *i* area, and *W* is the spatial weight matrix. Moran’s I is the Moran index, with a range of [–1,1]. When the Moran index is greater than 0, it indicates that the regional economic resilience has a spatially significant positive correlation; when the Moran index is less than 0, it indicates that the regional economic resilience has a spatially significant negative correlation; when the Moran index is equal to 0, it means that the regional economic resilience has no spatial correlation. The specific test results are shown in Table 2.

**Table 2:** Moran index test.

Variables	W_1_	W_2_	W_3_
2014	0.379***	0.125***	0.596***
2015	0.387***	0.125***	0.600***
2016	0.373***	0.120***	0.584***
2017	0.351***	0.108***	0.538***
2018	0.298***	0.094***	0.511***
2019	0.306***	0.099***	0.503***
2020	0.308***	0.100***	0.474***
2021	0.307***	0.100***	0.438***
2022	0.293***	0.097***	0.399***

#### 3.3.3. Spatial measurement model selection

To validate the use of which spatial effect model, first determine the adequacy of the SEM model, SAR model, and SDM model based on LM tests; then, through LR tests and WALD tests, further confirm whether the SDM model will degrade into SAR or SEM models. The specific test results are shown in Table 3.

**Table 3:** Spatial measurement model selection test.

TEST	W_1_	W_2_	W_3_
LM_err	1.128	5.817**	0.665
R-LM_err	0.046	3.211*	4.356**
LM_lag	2.669	3.442*	18.270***
R-LM_lag	1.587	0.835	21.962***
LR(SDM&SAR)	131.13***	33.98***	42.29***
LR(SDM&SEM)	162.28***	50.34***	71.59***
Wald	28.05***	11.56**	21.90***
	33.73***	16.97***	27.85***

LM-err test spatial autocorrelation error model, LM-lag test spatial autoregressive lag model; R-LM err and R-LM lag are supplements for stability test of Lagrange multiplier. The results indicate that the fixed effects of the spatial Durbin model are superior to the random effects, suggesting that fixed effects should be chosen for analysis. LR(SDM&SAR) indicates the test to see if the fixed effects of the Durbin model are superior to those of the spatial lag model, with results suggesting that the spatial Durbin model should be chosen. LR(SDM&SEM) tests whether the fixed effects of the Durbin model are superior to those of the spatial error model, with results also indicating the spatial Durbin model should be selected. Lastly, the Wald test is used to determine if the spatial Durbin model degenerates into the spatial lag model or the spatial error model. The results show that the spatial Durbin model does not degenerate into the spatial lag or spatial error models.

Based on the results in Table 2, this paper selects Spatial Durbin model (SDM):

$$RER_{it}=a_{0}+\rho_{1}WRER_{it}+a_{1}DIG_{it}+\rho_{2}WDIG_{it}+a_{x}Z_{it}+\rho_{3}WZ_{it}+\mu_{i}+d_{t}+\varepsilon_{it}$$

W is the spatial weight matrix, and ρ is the spatial autoregressive coefficient.

### 3.4. Threshold-effects model

Considering the potential nonlinear impact of digital industry development on regional economic resilience due to varying degrees of environmental regulation, we draw upon Hansen’s proposed threshold regression model to construct the following model [66]:

$$RER_{it}=a_{0}+a_{1}DIG_{it}•I(ENR\leq \lambda )+a_{2}DIG_{it}•I(ENR>\lambda )+a_{c}Z_{it}+\mu_{i}+d_{t}+\varepsilon_{it}$$

ENR Representation of the degree of environmental regulation, *λ* Representative threshold, *I*() Representative exponential function。

### 3.5. Data description

The dependent variable in this paper: Regional Economic Resilience (RER); the core explanatory variable in this paper: Digital Industries (DIG); the control variables in this paper: Transportation Accessibility (TRA), Energy Consumption (ENE), Industrial Structure (IST), Level of Marketization (MAR); the threshold variable in this paper: Environmental Regulation (ENV). Missing values in some parts are filled using interpolation method. The specific data description is shown in Table 4:

**Table 4:** Descriptive statistics.

Variable	Obs	Mean	Std. Dev.	Min	Max
RES	270	0.236	0.111	0.089	0.631
DIG	270	9.811	1.016	7.066	12.223
ENV	270	0.002	0.002	-0.001	0.028
TRA	270	0.998	0.527	0.101	2.363
ENE	270	0.353	0.141	0.001	0.659
IST	270	1.429	0.769	0.666	5.551
MAR	270	8.430	1.860	3.739	12.864

## 4. Empirical results

### 4.1. Benchmark regression

This article employs a two-way fixed-effects model for regression analysis, with specific results shown in Table 5.

**Table 5:** Baseline regression.

RES	Coef.	Robust Std. Err.	t	P>|t|	[95% Conf. Interval]	
Dig	0.024**	0.009	2.50	0.018	0.004	0.044
Tra	-0.076*	0.035	-2.13	0.042	-0.150	-0.003
Ene	-0.096	0.097	-0.99	0.332	-0.295	0.103
Ist	-0.019	0.017	-1.13	0.269	-0.054	0.015
Mar	0.013**	0.005	2.26	0.032	0.001	0.024
_CONS	-0.012	0.118	-0.11	0.917	-0.255	0.230

From Table 5, the development of the digital industry has a significant positive impact on regional economic resilience at the 5% level, indicating that the growth of the digital industry positively affects regional economic resilience. This result confirms hypothesis H1, which posits that the development of the digital industry can effectively enhance the level of regional economic resilience. This conclusion is supported by both theoretical literature and the practical situation in China. As a crucial driver for China’s economic recovery, the digital industry shows a trend of fluctuating growth during economic downturns, demonstrating strong industrial resilience and radiating effects, exhibiting counter-cyclical characteristics [67]. This is primarily due to the "moat effect" of the digital industry, which, through its reliance on self-innovation models, is less affected by other industries, making it easier to stabilize economic fluctuations and seek new development paths. However, as an exploratory analysis, while it provides valuable insights into the role of digital industry development in enhancing regional economic resilience, it also raises further questions. Within the analytical framework of evolutionary economic geography, spatial location plays an indispensable role in the study of regional economic resilience. The digital industry has shown a significant positive impact on the economic resilience of its local region, but whether it also has a spillover effect on adjacent and surrounding areas still requires further clarification.

### 4.2. Robustness and endogeneity testing

Before addressing the issue, further examination of the accuracy of the aforementioned experimental results is necessary. Firstly, considering the robustness issue of panel data, this paper adopts the method of excluding direct-controlled municipalities for regression analysis. Simultaneously, taking the ’Guiding Opinions on the Action of Internet Plus’ as an exogenous policy shock, a propensity score matching double difference method is used for regression [68]. Specific results are shown in columns (1) and (2) of Table 6. Meanwhile, considering the endogeneity issue of panel data, referring to Huang Qunhui et al., the interaction term between the number of post offices per million people in 1984 and the previous year’s national information technology service revenue is used as an instrumental variable [69]. Because the number of post offices per million people in 1984 is cross-sectional data, difficult to measure in panel data, following the setting method of Nunn et al., interaction terms are used for testing [70], and the results are shown in column (3) of Table 6. Finally, to avoid time discrepancies, this article has adjusted the sample period, shortening the investigation period to 2016–2022. The main reason for this change is that after the G20 Summit in 2016, China implemented the development of the digital economy as a strategic policy [71]. The specific results are shown in Column 3 of Table 6.

**Table 6:** Robustness and endogeneity testing.

RES	Excluding direct-controlled municipalities	PSM-DID	Instrumental variable	Time
DIG	0.026** (2.38)		0.088*** (5.16)	0.0237*** (2.79)
DID		0.002*** (6.69)		
TRA	-0.065 (-1.10)	0.041 (0.57)	-0.051 (-1.45)	-0.0953** (-2.03)
ENE	-0.093 (-0.92)	0.034 (0.55)	-0.078 (-0.76)	-0.0658 (-0.61)
IST	-0.042** (-2.64)	-0.042* (-1.97)	-0.009 (-0.67)	-0.0117 (-1.01)
MAR	0.011 (1.67)	0.002 (0.34)	0.011* (1.72)	0.0213*** (2.81)
_CONS	-0.035 (-0.26)	0.210 (1.74)	-1.02*** (-3.86)	-0.0585 (-0.54)

First, considering that municipalities directly under the central government differ from provinces in terms of city size, development models, etc., this could lead to regional data sample bias. Therefore, after excluding these municipalities, we conducted a regression analysis [72]. The results, as shown in Column 1 of Table 6, indicate that digital industries have a significant positive impact on regional economic resilience at the 5% level, with a coefficient of 0.026, thereby confirming Hypothesis H1.Secondly, to ensure that other characteristics of the treatment and control groups remain consistent before and after the policy implementation, this study employs the propensity score matching method to more accurately identify the impact of digital policies on regional economic resilience [73]. The results, shown in Column 2 of Table 6, indicate a positive coefficient at the 1% level, further confirming H1.Moreover, the reason for the endogeneity problem in economic variables could be due to two factors: first, endogeneity caused by omitted variables, which has been addressed by fixing time and individual fixed effects in the baseline regression. Second, there may be a causal relationship between digital industries and regional economic resilience; hence, this study uses instrumental variables to test for endogeneity issues [74]. As shown in Column 3 of Table 6, the study finds a significant positive impact of digital industries on regional economic resilience at the 1% level, confirming Hypothesis H1. Finally, the sample time deviation may lead to uncertainty in the research results. Therefore, this paper changes the sample period (2016–2022) to verify the accuracy of the results [75]. As shown in column 4 of Table 6, the regression coefficient is significantly positive and there is no numerical difference, indicating that even after changing the sample period, the digital industry can still promote the development of regional economic resilience, confirming that hypothesis H1 is valid.

### 4.3. Spatial Durbin model regression analysis

This text employs the spatial Doberman model for regression, and the specific results are as follows:

As shown in Table 7, under the adjacency, distance, and economic matrices, the spatial spillover effect of digital industry development on regional economic resilience is significant at the 1% level. Among these, the spatial spillover effect of the digital industry on regional economic resilience is greatest under the distance matrix. This result supports Hypothesis H2a, which posits that digital industry development has a positive spatial spillover effect on regional economic resilience. Next, we will analyze each case based on theoretical literature and practical situations. Under the adjacency matrix, the theoretical basis is that there is implicit knowledge transfer and demonstration effects among digital enterprises in neighboring regions [76]. Leveraging their platform and information dissemination characteristics, the learning costs for digital enterprises in neighboring regions decrease, further promoting innovation and development among these enterprises, thereby enhancing regional economic resilience. The practical basis is that China often groups adjacent provinces into the same economic development zones, such as the Beijing-Tianjin-Hebei region, the Yangtze River Delta, and the Chengdu-Chongqing economic belt. This results in more frequent economic exchanges and more convenient business interactions among these neighboring provinces, further strengthening the digital industry’s radiating effect on the economic resilience of adjacent regions [77]. Under the distance matrix, the development of the digital industry also shows a significant positive correlation with the regional economic resilience of both the local and surrounding areas. The possible reason lies in the accelerated development of the digital industry, which facilitates the flow of data elements across regions, strengthens the construction of data trading platforms, enhances resource utilization efficiency, and thus boosts regional economic vitality [78]. Under economic distance, the development of the digital industry can effectively empower the resilience of regional economies. This aligns not only with theoretical logic but also with the practical context in China. Theoretical logic suggests that due to similar business environments and levels of economic development in the regions where digital enterprises are located, there is a strengthened sense of corporate identity, which promotes interconnectivity and cooperation between enterprises, increases the frequency of information flow, and addresses issues like information asymmetry, thereby enhancing regional economic resilience [79]. The practical context is that China has consistently made common prosperity a national strategic goal. Large digital enterprises, leveraging advanced digital technologies, have launched "Actions to Support Common Prosperity" and actively assumed social responsibilities, thus promoting the improvement of resilience levels across different economic regions. In summary, the digital industry has a certain impact on improving the resilience level of regional economies within the context of spatial location.

**Table 7:** Spatial Doberman model regression.

Spatial Effect	W_1_	W_2_	W_3_
Direct Effect	0.008* (1.86)	0.011** (2.31)	0.022*** (4.28)
Indirect effect	0.082*** (12.07)	0.234*** (5.52)	0.067*** (5.47)
Overall Effect	0.091*** (13.71)	0.245*** (5.64)	0.089*** (6.29)

Note: ***, ** and *, indicating significant at 0. 01,0. 05 and 0. 1, respectively, with corresponding t-value in parentheses.

### 4.4. Resilience and digital industrialization: The perspective of environmental regulation

The focus of the aforementioned study lies in the direct impact of digital industry development on the resilience of regional economies. Subsequently, this article approaches the subject from the perspective of environmental regulations, examining the role of the level of environmental regulation in empowering the resilience of regional economies through the development of the digital industry. Policy influence plays a significant role in creating pathways within the region, thus understanding the evident role in regional economic resilience, seeking ways for economic breakthroughs. The stringency of environmental management and the increase in corporate costs have become an undeniable issue. Therefore, this paper treats environmental regulations as a threshold variable, studying the impact of digital industry development on regional economic resilience under different degrees of environmental regulation. The test results in Table 8 indicate that there is a single threshold for the improvement of regional economic resilience due to digital industry development, with the threshold value being 0.0011, as shown in Table 9.

**Table 8:** Results of the threshold-effects test.

Threshold	RSS	MSE	Fstat	Prob	Crit10	Crit5	Crit1
Single	0.1287	0.0005	27.06	0.000	10.950	13.291	17.179
Double	0.1219	0.0005	13.29	0.287	21.681	25.701	35.194
Triple	0.1181	0.0005	7.63	0.427	20.427	33.287	51.737

**Table 9:** Threshold value.

model	Threshold	Lower	Upper
Th-1	0.0011	0.0011	0.0011

As shown in Table 10, regardless of the level of environmental regulation, the development of the digital industry has a significantly positive impact on the enhancement of regional economic resilience. When the level of environmental regulation is below the threshold of 0.0011, the coefficient of the impact of digital industry development on regional economic resilience is 0.041; when the level of environmental regulation exceeds the threshold of 0.0011, the coefficient of the impact of digital industry development on regional economic resilience is 0.039. This empirical result is consistent with hypothesis H3, indicating that the impact of digital industry development on regional economic resilience exhibits a threshold effect of environmental regulation. When environmental regulation is at a lower threshold, the impact of digital industry development on enhancing regional economic resilience is greater; when environmental regulation is at a higher threshold, the impact of digital industry development on enhancing regional economic resilience is smaller. Analyzing the practical situation in China, firstly, many digital industry enterprises still face relatively backward technology and equipment, and the industrial structure is not very reasonable, which leads to higher energy consumption and environmental pollution issues. When the degree of environmental regulation exceeds the threshold range, the pollution control costs for these digital enterprises will rise, thus reducing the innovation costs for enterprises, consequently lowering the ability to enhance the resilience level of the regional economy. Secondly, the government is vigorously promoting the optimization of industrial structure and the transformation of digital enterprises, during which the consumption of energy costs is relatively high, and there is also a squeezing of enterprise innovation costs, thereby affecting the resilience of the regional economy. Thirdly, compared to traditional industries, the digital industry is more environmentally friendly, but tracing back to the upstream of the digital industry, pollution emissions from such enterprises may still exist. If environmental regulations are too stringent, it may lead to an increase in costs for upstream industries, which will then be passed on to digital enterprises, thereby affecting the resilience of the regional economy.

**Table 10:** Threshold-effect regression results.

RES	Coef.	Std. Err.	t	P>|t|	[95% Conf. Interval]	
TRA	-0.064***	0.022	-2.89	0.004	-0.109	-0.020
ENE	-0.177***	0.051	-3.48	0.001	-0.278	-0.077
IST	0.011	0.008	1.32	0.189	-0.005	0.029
MAR	0.027***	0.003	8.63	0.000	0.020	0.033
*DIG*•*I*(*ENR*≤*λ*)	0.041***	0.005	7.75	0.000	0.030	0.051
*DIG*•*I*(*ENR*>*λ*)	0.039***	0.005	7.37	0.000	0.028	0.049
_CONS	-0.269***	0.056	-4.79	0.000	-0.380	-0.158

In summary, the results of the hypotheses and arguments derived in this paper are presented in the table below, as shown in Table 11.

**Table 11:** Hypothetical argument result.

Hypothetical argument result	
H1	The development of the digital industry can effectively empower the enhancement of regional economic resilience.
H2a	The development of the digital industry has a positive spatial spillover effect on regional economic resilience.
H3	The impact of digital industry development on regional economic resilience exhibits a threshold effect due to environmental regulation. When environmental regulation is at a lower threshold, the impact of digital industry development on enhancing regional economic resilience is greater; when environmental regulation is at a higher threshold, the impact of digital industry development on enhancing regional economic resilience is smaller.

## 5. Research discussion and conclusion

### 5.1. Limitations of the study and directions for future research

The limitations of this article provide new research directions for this topic. Firstly, although this study explores the impact of spatial and geographic factors in the digital industry on regional economic resilience, there are significant differences in economic resilience among various regions in China due to factors such as geographic environment, development strategies, and regional positioning. However, this paper does not delve into the differences in time and space. Therefore, future research should focus on analyzing the development stages and spatial characteristics of different regions to gain a deeper understanding of how these factors significantly affect regional economic resilience. Secondly, in evaluating regional economic resilience, cultural systems, local policies, and social psychology across different regions all have significant impacts. However, since these factors are difficult to quantify and measure, this study did not consider them. Hence, it might be beneficial to conduct a more comprehensive interpretation of regional economic resilience by intersecting multiple disciplines, such as sociology, psychology, and management [80], thereby enriching the completeness of the regional economic resilience framework. In the end, although this study introduced the concept of regional economic resilience from the perspective of evolutionary economic geography, we still need to further refine the relevant theories to gain a deeper understanding of the true meaning and scope of regional economic resilience. This implies that we need to construct a more comprehensive and effective evaluation method based on dynamic changes and evolutionary mechanisms across different temporal and spatial scales, in order to develop a more scientific and complete evaluation index system.

### 5.2. Research conclusion

The research findings of this study are as follows: First, the development of China’s digital industry can effectively enhance regional economic resilience. Second, under different spatial matrices, the digital industry in China exhibits spatial spillover effects on regional economic resilience. Third, when the level of environmental regulation is below 0.0011, the development of China’s digital industry can more effectively enhance regional economic resilience. Based on the research findings, we provide the following research recommendations: First, attach importance to the role of the digital industry in promoting regional economic resilience. Governments should increase support for the digital industry, foster new industries through digital technology, promote the formation and development of the digital industry. At the same time, utilize the development of the digital industry to drive digital technology innovation, achieve the convergence of digital technology with a wide range of application scenarios, thereby effectively enhancing regional economic resilience. Secondly, build regional digital infrastructure to meet the development needs of the digital industry, cultivate the improvement of public digital literacy, avoid data monopolies and vicious competition among digital enterprises, and further stimulate regional economic vitality. Second, promote regional cooperation and development to achieve mutual benefit and win-win results. Actively explore regional cooperation mechanisms for the digital industry, remove administrative constraints and institutional barriers that affect the smooth flow of information resources between different regions, and reduce transaction costs. Secondly, based on regional development strategies and planning, construct a reasonable layout of the digital industry, fully leveraging its permeability and pioneering nature. By strengthening economic cooperation between regions, enhance the spillover effects of economic resilience, thereby boosting the collective resilience of both the local area and surrounding regions. Third, accelerate the digital enterprise transformation, optimize the industrial structure, and achieve green development. As the digital industry continues to transform, different solutions should be adopted for digital enterprises at various stages of transformation. Digital enterprises with irrational industrial structures and high energy consumption need to phase out outdated production capacities, introduce advanced production management technologies, and reduce pollution and emissions. For digital enterprises in transformation, it is necessary to strengthen their independent R&D capabilities, improve the efficiency of technology transfer, and achieve digital transformation. At the same time, the government should leverage the leading role of major digital companies to foster collaboration across various fields among digital enterprises, thereby creating new forms of modern digital industrial chains.

## Supporting information

S1 Data (ZIP)
